# Value of Life

**Published:** 1877-04

**Authors:** 


Mr. M. P. Levyeau is the authorized canvassing agent for the Journal. All contracts for advertisements made by him will be faithfully carried out by the publisher.

---

Value of Life.—The value of man. Dr. Farr made calculations of the value of man, as a producer from infancy up, that are both curious and interesting.

Basing it upon the agricultural classes of Norfolk, he estimated that an infant at birth was worth twelve dollars and a half, in its prospective labor. Five years later his value as a productive agent was one hundred and thirty dollars, and five years later it was more than doubled. At the age of twenty-five he has attained the maximum value, six hundred and fifteen dollars a year. At fifty it is reduced down to three hundred and forty-five dollars, and so on down to seventy, where the value is only two dollars less than nothing.—*Journal of Inebriety.*

